# Transcriptome Dynamics of Terminal Buds During Flower Bud Morphogenesis in Blueberry

**DOI:** 10.3390/genes17070837

**Published:** 2026-07-21

**Authors:** Xingyu Lu, Dongyu Sun, Yinyan Yang, Yalan Liu, Qin Yang, Biyan Zhou

**Affiliations:** 1 Provincial Famous Teacher Yang Qin Studio/Guizhou Key Laboratory of Molecular Breeding for Characteristic Horticultural Crops, College of Life and Health Science, Kaili University, Kaili 556011, China; luxingyu90@163.com (X.L.); lyl20250706@163.com (Y.L.); 2 College of Horticulture, South China Agricultural University, Guangzhou 510642, China; dy-sun@outlook.com (D.S.); 13202002376@163.com (Y.Y.)

**Keywords:** blueberry, floral bud morphogenesis, bud transcriptome, flowering regulation, co-expression network

## Abstract

**Background/Objectives**: Flower bud morphogenesis is a critical developmental phase during which blueberry transitions from vegetative to reproductive growth, yet the transcriptome dynamics and regulatory networks within buds during this process have not been systematically characterized. **Methods**: Terminal buds of the rabbiteye blueberry ‘Brightwell’ were sampled at six time points spanning from summer shoot cessation to bud swelling and dormancy entry. RNA sequencing, trend clustering, and Pearson correlation network analyses were performed to identify potentially important genes and regulatory relationships. **Results**: A total of 26,302 differentially expressed genes were identified, with a major transcriptomic shift at week 15. From four major expression trends, 1050 candidate genes were selected, including 176 flowering-related genes, 770 transcription factors, and 277 hormone-related genes. Photoperiod and vernalization pathway genes (*COLs*, *VRN1*, etc.) were predominantly down-regulated, whereas age pathway genes (*SPLs*) and MADS-box flower development genes (*FULs*, *AP3*, *PI*, and *SEP2*, etc.) were up-regulated. The floral integrators *FT* and *SOC1* exhibited opposite expression dynamics: *SOC1* was highly expressed during the early-to-mid stage, whereas *FT* peaked at the late stage, with the two showing opposing co-expression and correlation patterns. This suggests stage-specific divergence between the two integrators in coordinating flower bud differentiation and dormancy entry. Multiple hormone pathways (IAA, BR, JA, and SA, etc.) converged independently onto floral regulatory hubs through their biosynthesis/metabolism and signal transduction genes, with numerous transcription factors also involved. **Conclusions**: These findings provide a comprehensive view of bud transcriptome dynamics and propose a molecular regulatory framework integrating flowering signals, hormones, and transcriptional cascades, offering a theoretical foundation and gene resources for blueberry breeding.

## 1. Introduction

Blueberry (*Vaccinium* spp.) is a shrub native to North America. Owing to its distinctive fruit flavor and exceptional nutritional and health-promoting properties, the global cultivation area of blueberry continues to expand, establishing it as one of the most promising high-value small berries [1]. Flower bud formation constitutes a critical biological foundation for blueberry yield, as bud quantity and quality directly govern the capacity for flowering and fruit set in the following year. Unlike annual herbaceous plants, flower bud differentiation in perennial woody fruit trees such as blueberry is a protracted process. It sequentially undergoes physiological differentiation and early morphological differentiation before the flower buds enter dormancy, with the remaining floral organ differentiation and anthesis occurring the next year [2,3]. The flower bud morphogenesis stage is the core phase during which blueberry transitions from vegetative to reproductive growth and completes floral organ primordia differentiation. This stage begins with the cessation of summer shoot growth and ends when flower buds swell and enter dormancy. Therefore, elucidating the molecular regulatory mechanisms within bud tissues during this stage is crucial for understanding the flowering regulation and for manipulating the production cycle in blueberry.

In the model plant *Arabidopsis thaliana*, the flowering regulatory network has been extensively characterized. Upstream signals from the photoperiod, vernalization, age, autonomous, gibberellin, and ambient temperature pathways are integrated through multiple regulatory layers, ultimately converging on floral integrators such as *FLOWERING LOCUS T* (*FT*) and *SUPPRESSOR OF OVEREXPRESSION OF CONSTANS 1* (*SOC1*). These integrators subsequently activate downstream floral meristem identity genes, including *APETALA1* (*AP1*), *LEAFY* (*LFY*), and *FRUITFULL* (*FUL*), thereby initiating the floral organ developmental program [4,5]. Unlike annual plants, the flowering regulatory network in perennial woody species is often more complex [6]. In recent years, substantial progress has been made in understanding the regulation of flowering in blueberry. The group led by Song has systematically investigated chilling-mediated vernalization, photoperiod responses, and key flowering genes. Notably, overexpression of *VcFT* can partially substitute for the chilling and photoperiod requirements, promoting early and continuous flowering [7,8]. *VcFT* is highly expressed in leaves, and the synthesized FT protein is transported long-distance via the phloem and accumulates in buds to trigger the floral transition, activating downstream floral meristem identity genes such as *VcAP1*, *VcFUL*, and *VcLFY* to initiate flower primordia formation [9,10]. *VcSOC1* promotes flowering, and overexpression of its K-domain (*VcSOC1K*) in blueberry functions as a key activator of chilling-mediated bud break, significantly increasing flower bud number and yield potential [11,12]. Furthermore, the MIKC-type MADS-box gene family in blueberry was identified, and the expression peaks of genes such as *VcAP1.4*, *VcSVP9*, and *VcFLC2* during dormancy release were found to be associated with chilling accumulation [13]. The important roles of *SQUAMOSA PROMOTER BINDING PROTEIN-LIKE genes* (*SPLs*) like *VcSPL40*, *VcSPL35*, *VcSPL45*, and *VcSPL53* during the floral transition in blueberry were also highlighted [14].

The bud serves as the direct site of floral organ formation. The dynamics of gene expression within buds not only determine the progression and quality of flower bud differentiation but also integrate long-distance flowering signals (e.g., FT protein) from leaves and endogenous hormone signals to sequentially activate floral meristem identity and floral organ identity genes [15,16]. Hormonal signaling pathways are closely linked to the regulation of flowering in blueberry. Both *VcFT* and *VcSOC1K* overexpression induce differential expression of hormone-related genes [8,12]. Moreover, studies on the transgenic mutant *Mu-Legacy* have indicated that hormone pathway genes, such as *VcRR2*, may participate in chilling-mediated bud break by modulating gibberellin (GA) and cytokinin (CTK) signaling [17,18].

To date, there is a lack of systematic studies on the temporal expression dynamics, key regulatory modules, and their synergistic interplay with hormone signals at the whole-transcriptome level in blueberry buds during flower bud morphogenesis. Guizhou Province is a major blueberry-producing region in China, where climatic stresses such as low light, high humidity, and temperature fluctuations challenge flower bud differentiation [19]. ‘Brightwell’, the predominant rabbiteye cultivar in this region, is well adapted and high-yielding, making it a suitable subject for studying flower bud morphogenesis. Here, building on our previous leaf transcriptome analysis [20], we focus on buds as the terminal organs that perceive and integrate flowering signals. By establishing six intensive time points spanning the entire flower bud morphogenesis stage and employing high-throughput transcriptome sequencing, trend clustering, and Pearson correlation network analyses, we systematically identified flowering-related genes, transcription factors, and genes involved in hormone biosynthesis, metabolism and signal transduction. We constructed co-expression regulatory networks with an emphasis on the synergistic regulatory relationships among these gene categories. This study aimed to elucidate the molecular regulatory network in blueberry buds during flower bud morphogenesis, thereby providing a theoretical foundation and genetic resources for a deeper understanding of the flowering mechanism in blueberry.

## 2. Materials and Methods

### 2.1. Plant Materials and Sampling

Twelve healthy six-year-old field-grown ‘Brightwell’ plants with uniform growth vigor (approximately 1.5–2 m in height) were selected in this study. The plants were cultivated in strip planting beds (25 m long × 1 m wide × 0.8 m high) filled with a mixture of leaf mold, pine needle compost, and peat moss (4:3:2, *v*/*v*/*v*). The experimental site was located at the Excellent Agri-Forestry Talents Training Practice Base of Kaili University (26°31′5.92″ N, 107°51′23.18″ E).

During the flower bud morphogenesis stage, defined as the period from cessation of summer shoot growth to the onset of dormancy, with bud scales tightly closed and no visible swelling, terminal buds were collected from current-year shoots (which would bear flowers and fruits in the following year). Sampling was conducted from 14 July to 27 October 2023, during which the day length decreased from 13 to 14 h in mid-July to about 11 h in late October. Over this period, the average daily maximum temperature in the experimental plots decreased from approximately 30 °C to 20 °C, while the average daily minimum temperature dropped from around 20 °C to 10 °C. The initiation point (week 0) was set when summer shoots ceased growth and exhibited ‘black tips’ (aborted and blackened young leaf at the shoot apex). Subsequent samples were collected every three weeks thereafter, yielding six time points: week 0, 3, 6, 9, 12, and 15, with the final time point (week 15) corresponding to bud swelling and dormancy entry. At each time point, buds from four plants were pooled to constitute one biological replicate, and three biological replicates were established per time point, resulting in a total of 18 samples. All samples were immediately frozen in liquid nitrogen and stored at –80 °C until further use.

### 2.2. Library Construction and Sequencing Data Analysis

Total RNA was extracted from the 18 samples using the RNAprep Pure polysaccharide and polyphenol plant total RNA extraction kit (DP441, Tiangen Biotech, Beijing, China) following the manufacturer’s instructions. RNA concentration and purity were assessed using a NanoDrop 2000 spectrophotometer (Thermo Scientific, Waltham, MA, USA), and RNA integrity was evaluated using an Agilent 2100 Bioanalyzer system (Agilent Technologies, Santa Clara, CA, USA) with the RNA Nano 6000 Assay Kit. For each sample, 1 μg of total RNA was used as input for library construction with the Hieff NGS Ultima Dual-mode mRNA Library Prep Kit for Illumina (Yeasen Biotechnology, Shanghai, China). The main steps included mRNA enrichment using oligo(dT)-coated magnetic beads, fragmentation with Fragmentation Buffer, first-strand and second-strand cDNA synthesis, end-repair, A-tailing, adapter ligation, and size selection and purification using AMPure XP beads (Beckman Coulter, Pasadena, CA, USA). The final cDNA libraries were enriched by PCR, and their concentration and quality were verified using a Qubit 2.0 fluorometer and the Agilent 2100 Bioanalyzer system. Sequencing was performed on an Illumina NovaSeq platform (Illumina, San Diego, CA, USA). The raw data have been deposited in the China National Genomics Data Center (https://ngdc.cncb.ac.cn/ (accessed on 27 January 2026)) under BioProject accession PRJCA056943 and GSA accession CRA037787.

Raw reads were processed by removing adapters, low-quality sequences, and rRNA sequences to obtain clean data. The clean reads were aligned to the blueberry reference genome (https://www.vaccinium.org/analysis/49 (accessed on 18 June 2025)) [21] using HISAT2 [22]. Sample correlation analysis, principal component analysis (PCA), gene functional annotation, differential expression analysis, trend analysis, and transcription factor identification were performed on the online analytical platform of Genedenovo Biotechnology Co., Ltd. (Guangzhou, China) (https://www.omicshare.com/ (accessed on 23 March 2026)). Annotation was carried out against the Nr (NCBI non-redundant protein sequences), GO (Gene Ontology), and KEGG (Kyoto Encyclopedia of Genes and Genomes) databases. Gene expression levels were quantified as FPKM (fragments per kilobase of transcript per million mapped reads). Differentially expressed genes (DEGs) were identified using the R package DESeq2 with criteria of FDR < 0.05 and |log_2_FC| ≥ 1 [23]. Trend analysis was performed using the STEM (Short Time-series Expression Miner) algorithm with a threshold of fold change > 2, and genes in the major trends were subjected to KEGG functional annotation and transcription factor identification.

### 2.3. Identification and Expression Analysis of Candidate Genes Potentially Important for Flower Bud Morphogenesis

Candidate genes with potential importance were screened from the genes belonging to the four major expression trends, with a focus on flowering-related genes, hormone biosynthesis and metabolism-related genes, hormone signal transduction-related genes, and genes encoding transcription factors (TFs). The screening was primarily based on KEGG annotation results, and additionally referenced the multi-species flowering gene regulatory network database (http://pfgd.bio2db.com/ (accessed on 6 April 2026)) [24] and the multi-species plant hormone biosynthesis, metabolism, signal transduction, and transport gene database (http://phgd.bio2db.com/ (accessed on 11 April 2026)) [25] developed by Song et al. Heatmaps were generated using TBtools software (version 2.154) [26] to visualize the expression patterns of flowering-related, hormone biosynthesis and metabolism-related, and hormone signal transduction-related genes.

### 2.4. Construction of Gene Co-Expression Networks

Given the excessively large number of DEGs between the 15-week time point and other time points, which would lead to highly uneven module sizes in WGCNA, Pearson correlation coefficients were used to evaluate co-expression relationships among genes. To reduce the influence of skewed data distribution and better reflect fold changes, FPKM values were log_2_-transformed (log_2_(FPKM+1)), and gene pairs with |r| ≥ 0.95 were selected for co-expression network construction. Network visualization was performed using Cytoscape software (version 3.9.0). Separate networks were constructed to display the associations of floral induction-related genes, floral integrators, flower development-related genes with transcription factors, hormone biosynthesis/metabolism and signal transduction genes, as well as the interconnections among these three categories of flowering-related genes. In the networks, orange and green lines indicate positive and negative correlations, respectively, and node size represents connectivity (degree value), with larger nodes indicating more interactions with other genes in the network.

### 2.5. Quantitative Real-Time PCR Validation

A subset of differentially expressed genes was randomly selected for validation by quantitative real-time PCR (qPCR). Total RNA was extracted from the backup samples using the same method as described in Section 2.2. For each sample, 1 μg of total RNA was reverse-transcribed into cDNA using the Hifair^®^ II 1st Strand cDNA Synthesis Kit (gDNA digester plus, Yeasen Biotechnology, Shanghai, China). Primers were designed using Primer Premier 5.0 software, with *ACTIN* used as the internal reference gene [27]; primer sequences are listed in Table S1. qPCR reactions were performed on a CFX384 Touch Real-Time PCR Detection System (Bio-Rad, Hercules, CA, USA) using Hieff^®^ qPCR SYBR Green Master Mix (No Rox, Yeasen Biotechnology, Shanghai, China). The thermal cycling program was: 95 °C for 5 min; followed by 40 cycles of 95 °C for 5 s, 58 °C for 20 s, and 72 °C for 20 s. Each sample was run in triplicate, and relative expression levels were calculated using the 2^−ΔΔCT^ method [28].

### 2.6. Statistical Analysis

Statistical analysis was performed using SPSS software (version 19.0; IBM Corp., Armonk, NY, USA). One-way analysis of variance (ANOVA) followed by Duncan’s multiple range test (*p* < 0.05) was used to assess significant differences among time points. Pearson correlation coefficients were calculated to evaluate the consistency between qRT-PCR and RNA-Seq expression data for the validated genes.

## 3. Results

### 3.1. Transcriptome Sequencing Quality Assessment

**Figure 1:**
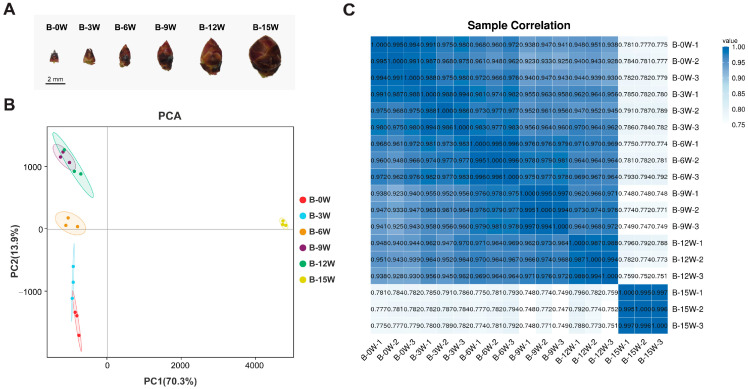
Bud morphology, principal component analysis, and correlation analysis of samples during the flower bud morphogenesis stage in blueberry. (**A**) Bud morphology at the six sampling time points. (**B**) PCA plot based on all expressed genes. (**C**) Heatmap of correlation coefficients among the 18 samples.

Bud samples from six time points during the flower bud morphogenesis stage in blueberry (Figure 1A) were used for library construction and high-throughput sequencing. A total of 129.93 Gb of raw data were generated, with individual libraries ranging from 5.85 to 11.23 × 10^9^ bp. After removing adapters, redundant reads, low-quality reads, and rRNA sequences, each library yielded 3.66–6.90 × 10^7^ clean reads, accounting for over 87% of raw reads, and the mapping rates against the blueberry reference genome were all above 81%, indicating high data quality and alignment efficiency (Table 1). Correlation analysis and principal component analysis (PCA) of the 18 libraries (Figure 1B,C) revealed good within-group biological reproducibility and high sample similarity. PCA showed clear separation among four groups: 0W/3W, 6W, 9W/12W, and 15W, with the 15W samples being the most divergent, suggesting that the gene expression profile at this time point differed substantially from those at other time points.

### 3.2. Identification of Differentially Expressed Genes

**Figure 2:**
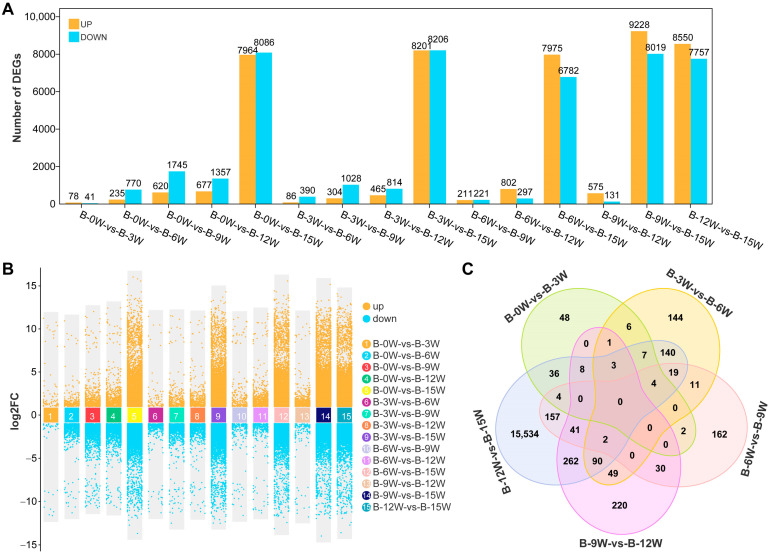
Screening of differentially expressed genes in bud transcriptomes during flower bud morphogenesis in blueberry. (**A**) Statistics of DEG numbers in each comparison group (|log_2_FC| ≥ 1, FDR < 0.01). (**B**) Volcano plots of DEGs. (**C**) Venn diagram showing the union of DEGs from adjacent time point comparisons.

A total of 26,302 differentially expressed genes (DEGs) were identified by DESeq2 analysis. As shown in Figure 2A,B, the number of DEGs between any two time points from 0W to 12W remained relatively low; pairwise comparisons between adjacent time points (0W vs. 3W, 3W vs. 6W, 6W vs. 9W, and 9W vs. 12W) yielded 119, 476, 432, and 706 DEGs, respectively. In contrast, the 15W time point, corresponding to rapid bud swelling and dormancy entry with pronounced phenotypic changes, exhibited a dramatic increase in DEGs when compared with each of the other time points (16,050, 16,407, 14,757, 17,247, and 16,307 DEGs against 0W, 3W, 6W, 9W, and 12W, respectively). Based on these data characteristics and the progression of flower bud differentiation and development, the union of DEGs from adjacent time-point comparisons (0W vs. 3W, 3W vs. 6W, 6W vs. 9W, 9W vs. 12W, and 12W vs. 15W) was taken, yielding 16,980 DEGs for subsequent analyses (Figure 2C).

### 3.3. Trend Analysis and KEGG Functional Enrichment of DEGs

**Figure 3:**
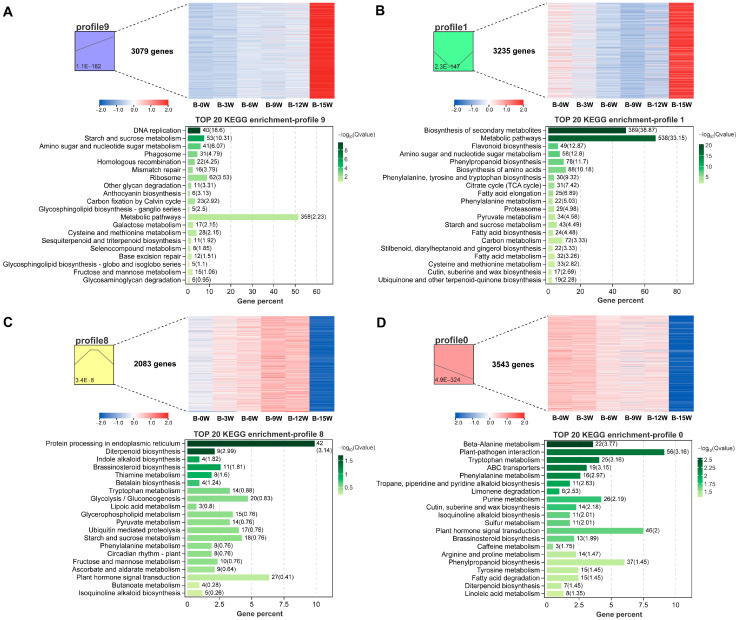
KEGG functional annotation of genes in the four major expression trends. (**A**) Top 20 enriched pathways for the continuously up-regulated trend (profile 9). (**B**) Top 20 pathways for the down-then-up trend (profile 1). (**C**) Top 20 pathways for the up-then-down trend (profile 8). (**D**) Top 20 pathways for the continuously down-regulated trend (profile 0). The values to the right of each bar indicate the number of genes annotated to that pathway and the corresponding *p*-value.

The expression profiles of the 16,980 DEGs were subjected to trend analysis, which identified four major expression trends: continuously up-regulated (profile 9), down-then-up (profile 1), up-then-down (profile 8), and continuously down-regulated (profile 0). The continuously up-regulated trend comprised 3079 genes, which were enriched in pathways such as DNA replication, starch and sucrose metabolism, and amino sugar and nucleotide sugar metabolism, with the largest number annotated to metabolic pathways (Figure 3A). The down-then-up trend included 3235 genes, enriched in biosynthesis of secondary metabolites, metabolic pathways, flavonoid biosynthesis, and other pathways, with biosynthesis of secondary metabolites and metabolic pathways having the highest gene counts (Figure 3B). The up-then-down trend contained 2083 genes, enriched in protein processing in endoplasmic reticulum, diterpenoid biosynthesis, indole alkaloid biosynthesis, and other pathways, with relatively abundant genes in protein processing in endoplasmic reticulum and plant hormone signal transduction (Figure 3C). The continuously down-regulated trend comprised 3543 genes, enriched in beta-alanine metabolism, plant–pathogen interaction, and tryptophan metabolism, with plant–pathogen interaction, plant hormone signal transduction, and phenylpropanoid biosynthesis containing the largest numbers of genes (Figure 3D). Overall, the most pronounced fluctuations in gene expression occurred at the 15W time point, with the majority of genes being sharply up- or down-regulated between 12W and 15W, while changes before that were relatively moderate.

### 3.4. Identification and Expression Pattern Analysis of Potentially Important Genes

From the four major trends, a total of 1050 potentially important genes involved in flower bud morphogenesis were identified, including 176 flowering-related genes (floral induction, floral integrators, and flower development), 770 genes encoding transcription factors (TFs), 140 hormone biosynthesis and metabolism genes, and 137 genes related to hormone signal transduction.

**Figure 4:**
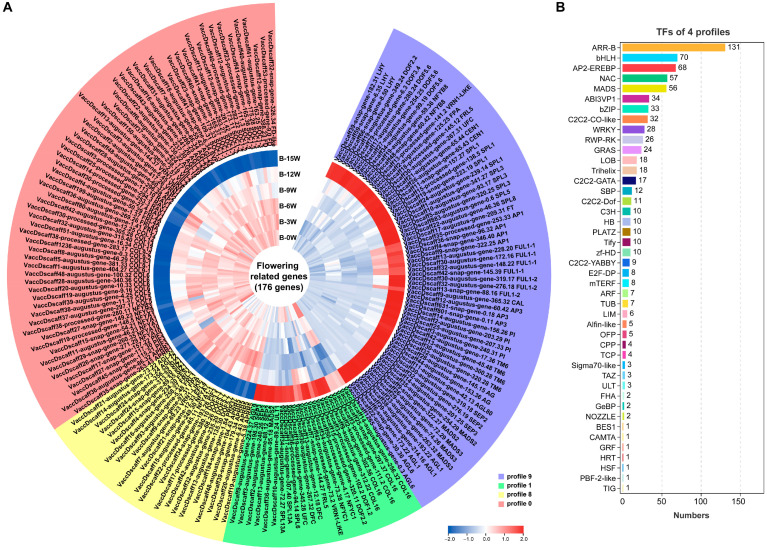
Expression heatmaps of flowering-related genes and transcription factor identification in the four major trends. (**A**) Heatmap of flowering-related gene expression. (**B**) Transcription factor families and member counts identified in the four trends.

Among the flowering-related genes, the continuously up-regulated (profile 9) and continuously down-regulated (profile 0) trends contained the largest numbers, each with 65 genes, whereas the down-then-up (profile 1) and up-then-down (profile 8) trends had only 23 genes each (Figure 4A). Notably, nearly all flower development-related genes belonged to the continuously up-regulated category, with only a very small number of genes showing the down-then-up pattern, and none were found in the continuously down-regulated or up-then-down categories. Within the continuously up-regulated group (profile 9), 25 floral induction-related genes were identified, mainly involving age pathway *SPLs* (*SPL1/3/5/8*) and circadian clock and photoperiod pathway genes (*LHY*, *NFYB8*, and *DOFs* such as *DOF2.2/3/4/5.6*), along with a few vernalization pathway-related genes. One floral integrator gene, *FT*, and 39 flower development-related genes (*AP1*, *FUL1-1/1-2*, *CAL*, *AP3*, *TM6*, *PI*, *AG*, *SEP2/3,* etc.), were also present. The down-then-up trend (profile 1) mainly comprised floral induction-related genes, such as circadian clock/photoperiod genes (*COL16*, *NFYC1*, *DOF1.2/2.2*), vernalization genes (*VRN1-LIKE*, *FRL5*, *DFC*, *UFC*), and age pathway genes (*SPL6/13A*), in addition to six flower development-related genes (*ULT1*, *SEP2/3*, etc.). Among the 65 continuously down-regulated genes (profile 0), 28 were involved in the circadian clock and photoperiod pathway, including 16 *CONSTANS-LIKE (COL)* genes (*COL5/6/9-1/9-2/13/14*) as well as *APRR1*, *PIF3*, *NFYA1/10*, *NFYB3*, and *NFYC9*; 23 genes were vernalization-related, including *VRN1*, *VRN1-LIKE*, *VIL2*, *FRL1*, *FRL4A*, *AGL19*, and *SVP*; additionally, 14 floral integrators were identified, comprising four *SOC1-1*, four *SOC1-2*, three *SOC1-3*, and three *FD* genes.

Transcription factor analysis (Figure 4B) showed that the 770 transcription factors belonged to 44 families, with ARR-B, bHLH, AP2-EREBP, NAC, MADS, ABI3VP1, bZIP, C2C2-CO-like, WRKY, and RWP-RK being the top 10 most abundant families.

**Figure 5:**
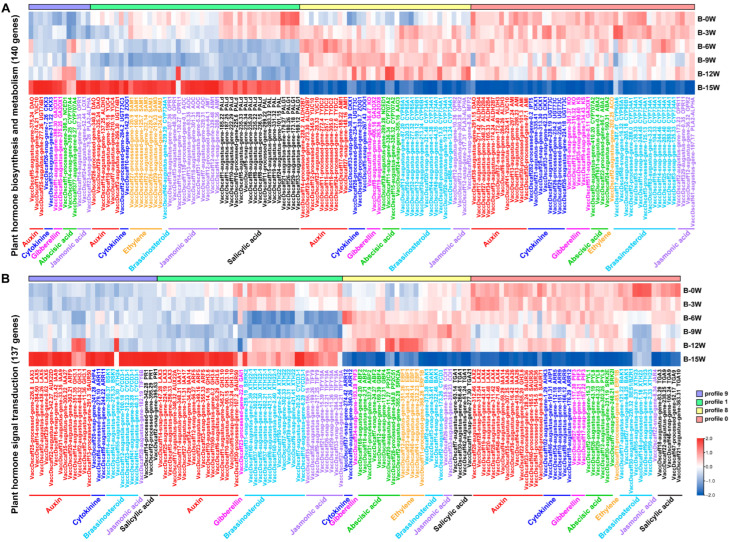
Expression heatmaps of hormone biosynthesis and metabolism-related genes (**A**) and hormone signal transduction-related genes (**B**) in the four major trends.

Hormone biosynthesis and metabolism-related genes covered eight hormone classes: auxin (IAA), cytokinin (CTK), gibberellin (GA), abscisic acid (ABA), ethylene (ETH), brassinosteroid (BR), jasmonic acid (JA), and salicylic acid (SA) (Figure 5A). The continuously up-regulated trend contained 13 genes, including those related to IAA, CTK, GA, ABA, and JA; the down-then-up trend had 44 genes covering IAA, CTK, ETH, BR, JA, and SA; the up-then-down trend included 36 genes related to IAA, CTK, GA, ABA, and BR; and the continuously down-regulated trend had 47 genes spanning IAA, CTK, GA, ABA, ETH, BR, and JA. Overall, among hormone-related genes showing sharp up-regulation at 15W (profiles 9 and 1), SA, JA, and IAA metabolism genes predominated, with 17, 14, and 9 members, respectively; among those sharply down-regulated at 15W (profiles 8 and 0), BR, IAA, and CTK-related genes were most abundant, with 24, 22, and 12 members, respectively.

For the hormone signal transduction-related genes (Figure 5B), the continuously up-regulated trend included 27 genes involved in IAA, CTK, BR, JA, and SA pathways; the down-then-up trend had 39 genes related to IAA, GA, BR, and JA; the up-then-down trend contained 27 genes implicated in CTK, GA, ABA, ERF, BR, JA, and SA; and the continuously down-regulated trend comprised 43 genes associated with IAA, CTK, GA, ABA, ERF, BR, and JA. Among hormone signal transduction genes sharply up-regulated at 15W, IAA and BR pathway genes were predominant (30 and 20, respectively), whereas those sharply down-regulated were dominated by IAA and ABA pathway genes (15 each).

### 3.5. Validation of Transcriptome Data by qRT-PCR

**Figure 6:**
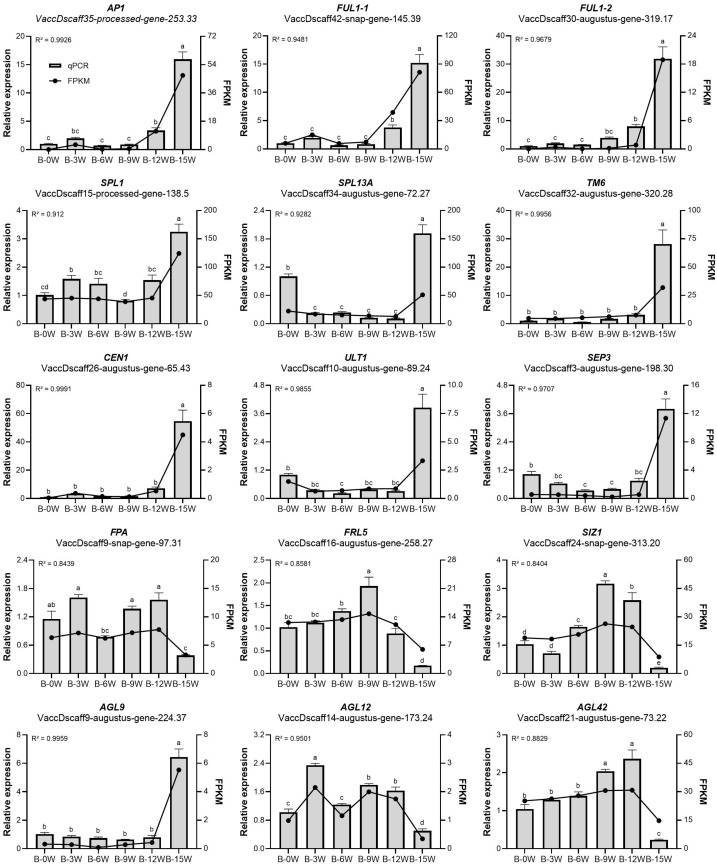
Validation of randomly selected genes by quantitative real-time PCR. The left y-axis corresponds to qRT-PCR results, and the right y-axis corresponds to RNA-Seq FPKM values. The R^2^ value in the upper-left corner of each panel indicates the correlation coefficient between the two datasets. qRT-PCR data are presented as the means of three biological replicates, with error bars representing standard errors (SE). Different lowercase letters denote significant differences among time points within each gene (*p* < 0.05, Duncan’s multiple range test).

Fifteen flowering-related genes were randomly selected for qRT-PCR validation. As shown in Figure 6, the expression trends obtained by qRT-PCR were generally consistent with the FPKM values from RNA-Seq. Correlation analysis between qRT-PCR and RNA-Seq results for each validated gene yielded R^2^ values greater than 0.84, confirming the reliability of the transcriptome data.

### 3.6. Co-Expression Network Construction of Potential Important Genes in Flower Bud Morphogenesis

**Figure 7:**
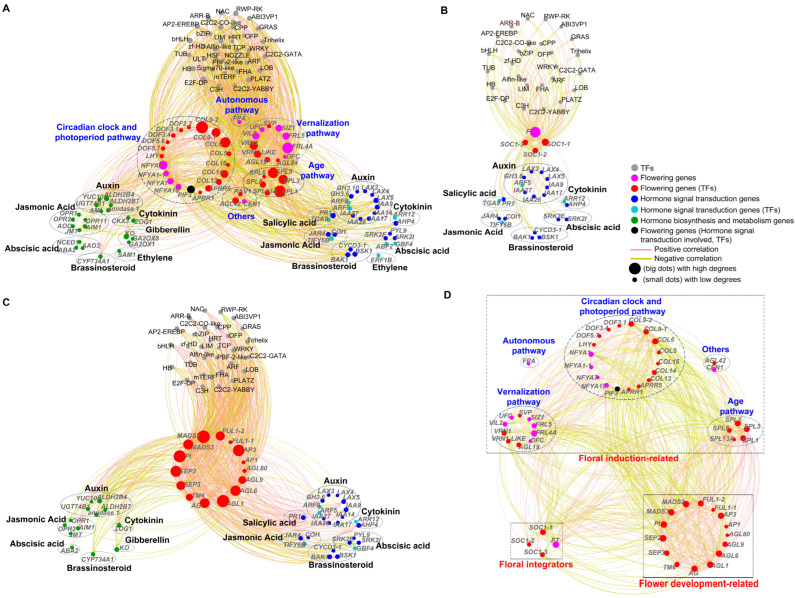
Co-expression networks of potentially important genes during flower bud morphogenesis stage in blueberry (correlation coefficient threshold |r| ≥ 0.95). (**A**) Co-expression network of floral induction-related genes with hormone biosynthesis/metabolism, hormone signal transduction, and transcription factor genes. (**B**) Co-expression network of floral integrators with hormone biosynthesis/metabolism, hormone signal transduction, and transcription factor genes. (**C**) Co-expression network of flower development-related genes with hormone biosynthesis/metabolism, hormone signal transduction, and transcription factor genes. (**D**) Co-expression network among floral induction-related genes, floral integrators, and flower development-related genes.

Based on the 1050 potentially important genes, a Pearson correlation matrix was calculated to construct co-expression networks, with emphasis on the associations of floral induction genes, floral integrators, and flower development genes with TFs, hormone biosynthesis/metabolism genes, and hormone signal transduction genes, as well as the interconnections among the three categories of flowering genes (Figure 7, Table S2).

As shown in Figure 7A, transcription factors, hormone biosynthesis/metabolism genes, and hormone signal transduction genes each showed strong correlations with floral induction-related genes, but no direct correlations were observed among these three categories themselves. The floral induction-related genes that exhibited connections with other genes included those involved in the circadian clock/photoperiod pathway (*COLs*, *DOFs*, *NFYAs*, *APRRs*, *PIF3*, *LHY*), vernalization pathway (*FRL4A/5*, *VRN1*, *VIL2*, *AGL19*, *SVP*, etc.), age pathway (*SPL1/3/5/6/8/13A*), autonomous pathway (*FPA*), and other related genes (*CEN1*, *RAV1*, *AGL42*), with the circadian clock/photoperiod pathway genes being the most numerous. The hormone biosynthesis and metabolism genes associated with floral induction genes included those of IAA (*ALDH2B4*, etc.), CTK (*CKX3*, etc.), GA (*KO*, etc.), ABA (*NCED1*, etc.), ETH (*SAM1*), BR (*CYP734A1*), and JA (*JMT*, etc.). Hormone signal transduction genes involved included those from IAA (*LAX5*, etc.), CTK (*AHP4*, etc.), ABA (*SRK2E*, etc.), ETH (*ERF1B*), BR (*BSK1*, etc.), JA (*JAR4*, etc.), and SA (*PR1*, etc.) pathways. The transcription factors belonged to 38 families, such as bHLH, ARR-B, AP2-EREBP, C2C2-CO-like, and ABI3VP1. Nodes with high connectivity included the photoperiod-pathway gene *COL9-2* (VaccDscaff38-processed-gene-283.17), the age pathway gene *SPL1* (VaccDscaff15-processed-gene-138.5), and the vernalization-pathway gene *FRL4A* (VaccDscaff36-augustus-gene-269.26).

The co-expression network of flower development-related genes (Figure 7C) exhibited a similar pattern, with the three categories showing strong correlations with flower development-related genes but no direct interconnections among themselves. Key flower development-related genes included *AP1*, *FUL1-1/1-2*, *MADS2/3*, *AP3*, *AG*, *TM6*, *SEP2/3*, *PI*, and *AGL1/6/9/80*. Associated hormone biosynthesis and metabolism genes encompassed IAA (*ALDH2B4*, etc.), CTK (*ZOG1*), GA (*KO*), ABA (*ABA2*), BR (*CYP734A1*), and JA (*OPR2*, etc.); hormone signal transduction genes involved IAA (*LAX5*, etc.), CTK (*AHP4*, etc.), ABA (*SRK2E*, etc.), BR (*BSK1*, etc.), JA (*JAR4*, etc.), and SA (*PR1*). Transcription factors included 30 families, with MADS family being the most abundant (38 members). Highly connected nodes were *MADS2* (VaccDscaff35-augustus-gene-254.29), *AGL1* (VaccDscaff38-augustus-gene-193.22), *PI* (VaccDscaff2-augustus-gene-203.29), *AP3* (VaccDscaff631-snap-gene-0.18), and *SEP2* (VaccDscaff32-augustus-gene-276.19).

In the network of floral integrators (Figure 7B), genes associated with *FT* and *SOC1-1/-1-2/-1-3* included hormone signal transduction genes predominantly from the IAA pathway (LAX3/4/5, IAA9/17/26/27, *ARF5*, etc.), with additional involvement from CTK (*AHP4*, *ARR12*), ABA (*SRK2E*, *SRK2I*), BR (*BSK1*, *BAK1*, *CYCD3-1*), JA (*JAR4*, *COI1*, *TIFY6B*), and SA (*PR1*, *TGA9*) pathways. Transcription factors encompassed 32 families, including bHLH, ARR-B, AP2-EREBP, RWP-RK, and ABI3VP1. Highly connected hub genes were *FT* (VaccDscaff17-augustus-gene-209.31) and *SOC1-1* (VaccDscaff40-snap-gene-175.33).

The interaction network among floral induction-related genes, floral integrators, and flower development-related genes (Figure 7D) showed that the most highly connected genes were *SEP2* (VaccDscaff32-augustus-gene-276.19), *PI* (VaccDscaff2-augustus-gene-203.29), and *FT* (VaccDscaff17-augustus-gene-209.31). Among floral induction-related genes, circadian clock/photoperiod pathway genes were predominant, with numerous *COL* family members exhibiting high connectivity; the vernalization gene *FRL4A* and the age pathway genes *SPL1* and *SPL3* also showed high connectivity. *FT* was negatively correlated with the three *SOC1* genes (*SOC1-1/-1-2/-1-3*), while flower development-related genes were all positively correlated with each other. Among these categories, circadian clock/photoperiod and vernalization pathway genes were mainly negatively correlated with flower development-related genes, whereas age pathway genes were all positively correlated with flower development-related genes. *SOC1* genes were predominantly positively correlated with circadian clock/photoperiod and vernalization genes, but negatively correlated with age pathway and flower development-related genes. Conversely, *FT* showed the opposite pattern, being mainly negatively correlated with circadian clock/photoperiod and vernalization genes, and positively correlated with age pathway and flower development-related genes.

## 4. Discussion

Flower bud differentiation is a critical biological process determining blueberry yield and serves as a central hub in the annual growth cycle [3]. The bud, as the direct site of floral organ formation, undergoes transcriptional dynamics that dictate the progression and quality of flower bud differentiation. In this study, through temporal transcriptomic profiling of bud tissues at six time points spanning the flower bud morphogenesis stage, we systematically identified potentially important genes involved in floral induction, flower development, hormone signaling, and transcriptional regulation, and constructed co-expression regulatory networks.

### 4.1. The Late Stage of Flower Bud Morphogenesis Is a Critical Node of Extensive Transcriptomic Reprogramming in Buds

Differential expression analysis revealed that the number of DEGs between adjacent time points from 0 to 12 weeks remained relatively low, indicating that this period is characterized by gradual transcriptional adjustments. However, at 15 weeks, when buds swelled rapidly and entered dormancy, the number of DEGs between this time point and each of the others surged to over 14,000 (Figure 2). This trend is highly consistent with our previous transcriptomic results from leaves during the same period [20], collectively confirming that the late stage of flower bud morphogenesis represents a critical window during which the entire plant undergoes a dramatic physiological transition. The clear separation trajectory of samples in the PCA further supports this conclusion (Figure 1B), showing a sharp shift in the gene expression profile at 15W. KEGG enrichment analysis showed that continuously up-regulated and down-then-up genes were significantly enriched in DNA replication, starch and sucrose metabolism, and various secondary metabolic pathways such as flavonoid biosynthesis (Figure 3A,B). This coincides with the rapid cell division and surging energy demand characteristic of flower bud swelling [29,30,31]. As the direct site of floral organ differentiation, bud tissues exhibited more pronounced transcriptional changes than leaves, underscoring their central role in the terminal response to floral signals and the establishment of floral primordia.

### 4.2. Temporal Expression and Synergistic Regulatory Networks of Flowering Genes

The expression patterns of flowering-related genes revealed a clear hierarchical regulatory structure in blueberry buds. At the floral induction level, a large number of genes exhibited continuously down-regulated trends (profile 0), including multiple *COL* genes involved in the photoperiod pathway (e.g., *COL5/6/9-1/9-2/13/14*) and vernalization pathway genes (e.g., *VRN1*, *VIL2*, *SVP*) (Figure 4A). The *COL* family is a key component of the photoperiod pathway, influencing flowering time through regulation of *FT* expression in *Arabidopsis* [32]. In rabbiteye blueberry, down-regulation of *VcCOL5* is associated with off-season flowering [33]. In this study, only *COL16* showed a down-then-up pattern, consistent with observations in leaf transcriptomes [20], suggesting that *COL16* may serve a function distinct from other members in the photoperiod response of blueberry flowering. In contrast, *SBP* family genes from the age pathway (*SPL1/3/5/8*) were continuously up-regulated. Previous studies have demonstrated that *VcSPL40*, *VcSPL35*, *VcSPL45*, and *VcSPL53* play important roles during the floral transition in blueberry [14]. Furthermore, compared with non-chilled buds, buds that had undergone chilling and dormancy release showed up-regulation of *VcCOL5/10* and *VcSVP*, down-regulation of *VcVIL2*, up-regulation of *VcSPL8/14*, and down-regulation of *VcSPL1/5/6/9/16* [27], indicating that floral induction-related genes exhibit distinct expression patterns between the morphogenesis stage and dormancy release.

Flower development-related genes were almost exclusively enriched in the continuously up-regulated trend (profile 9), including MADS-box genes such as *AP1*, *FUL1-1/1-2*, *AP3*, *PI*, *AG*, and *SEP2/3* (Figure 4A), consistent with their core functions in floral organ primordia differentiation. In *Arabidopsis*, multiple MADS-box members participate in floral organogenesis and development [34]. For instance, *AP1* and *FUL* are key determinants of floral meristem identity, regulating flowering time and floral organ developmental fate [35,36], while AP3-PI/SEP-SEP and AP3-PI/AG-SEP protein tetramers respectively regulate petal and stamen formation [37]. *AG* plays a central role in specifying stamen and carpel identity and meristem determinacy, and AG–SEP3 heterodimeric complexes regulate distinct target genes through differential binding sites, with direct targets causing early flowering and curled leaf phenotypes [38]. In the present study, these genes remained highly expressed at week 15, indicating that floral organ primordia differentiation in blueberry may continue until just before flower buds swell and enter dormancy. The expression peaks of *VcAP1.4*, *VcAP3.1*, and *VcAG3* were also found to be closely associated with dormancy release and flowering initiation [13]. The high connectivity of MADS-box genes in co-expression networks (Figure 7C) further confirms the central driving role of this family in floral organ primordia differentiation.

The expression patterns of floral integrators are particularly noteworthy. In *Arabidopsis*, *FT* and *SOC1* are key nodes connecting upstream signals with downstream floral developmental programs [5]. Functional studies in blueberry have confirmed that overexpression of *VcFT* and *VcSOC1K* both promote flower bud formation, albeit with differences in their stage of action and regulatory targets [7,12]. In leaves, an increased *VcFT/VcSOC1* ratio promotes flower bud initiation, whereas a decreased ratio in mature buds favors bud break after chilling fulfillment [27]. In our study, three *SOC1* genes (*SOC1-1/1-2/1-3*) maintained relatively high expression levels during the early-to-mid morphogenesis stage (0–12W), but their expression dropped sharply at 15W when buds swelled and were about to enter dormancy. In contrast, *FT* showed low expression from 0W to 12W but increased sharply at 15W. Co-expression analysis further revealed that *FT* was negatively correlated with the three *SOC1* genes. Notably, *SOC1* was predominantly positively correlated with circadian clock/photoperiod and vernalization genes, but negatively correlated with age pathway *SPLs* and downstream flower development genes (*FULs*, *AP3*, *PI*, and *SEP2*, etc.), whereas *FT* exhibited a reversed correlation pattern with these gene sets (Figure 7D). This contrasting temporal pattern, together with their opposing correlation patterns in the co-expression network, suggests that *FT* and *SOC1* may perform distinct and potentially antagonistic functions in buds during flower bud morphogenesis. Specifically, *SOC1* was highly expressed and positively correlated with photoperiod/vernalization signals during the early-to-mid stage (0–12W), when flower bud differentiation is actively progressing. In contrast, *FT* was expressed at low levels during this period but surges at 15W, when buds undergo rapid morphological changes and enter dormancy, and was positively correlated with downstream flower development genes. This temporal separation, with *SOC1* active in the differentiation phase and *FT* peaking at the transition to dormancy, points to a functional relay rather than simultaneous cooperation. Their negative correlation in the network further supports the notion of potentially opposing regulatory roles in the bud. Additionally, the high expression of *FT* detected in buds may originate from localized transcription within the bud tissue itself, occurring in boundary cells of the floral primordium independently of leaves [39].

### 4.3. Coordinated Regulation by Hormone Networks and Transcription Factors

Hormone signaling networks are highly active in regulating flowering in buds [16,40]. In our study, at the 15W time point, hormone-related genes exhibited pronounced differential changes at both the biosynthesis/metabolism and signal transduction levels. At the biosynthesis and metabolism level, sharply up-regulated genes were predominantly associated with SA, JA, and IAA, while sharply down-regulated genes were mainly related to BR, IAA, and CTK (Figure 5A). This trend suggests that the bud may prepare for floral bud morphological finalization and dormancy initiation by activating defense/development-related pathways such as SA and JA while gradually shutting down BR and CTK biosynthesis, which promote cell division and expansion. The sharp late-stage up-regulation of SA and JA biosynthetic genes is not only associated with defense responses preceding dormancy but may also be closely linked to floral organ development. SA participates in stamen and pistil development regulation [41], and JA has been reported to play positive roles in stamen development and anther formation [42], suggesting that these two hormones may participate in the late-stage floral organogenesis of blueberry. At the signal transduction level, up-regulated genes were enriched in IAA and BR pathways, whereas down-regulated genes were dominated by IAA and ABA pathways (Figure 5B). Notably, the BR pathway exhibited a divergent pattern of down-regulated biosynthesis but up-regulated signal transduction, implying that buds do not simply withdraw from BR signaling. Instead, they may up-regulate signal-responsive components to maintain sensitivity to residual signals under reduced BR biosynthesis, thereby precisely completing the final morphogenesis of floral organs [43]. Our previous leaf transcriptomic study also found that BR signaling pathway genes were the most abundant in blueberry leaves [20], further supporting the active participation of BR in flower bud morphogenesis. The IAA pathway exhibited both up- and down-regulated genes at the biosynthetic and signaling levels, reflecting that auxin signaling at this stage likely undergoes multi-directional changes rather than a simple overall increase or decrease.

Co-expression network analysis further linked these hormonal signals to the flowering regulatory framework. Floral induction genes, floral integrators, and flower development genes all formed strong associations with multiple hormone biosynthesis, metabolism, and signal transduction genes; however, neither hormone biosynthetic and signaling pathways nor different hormone classes were directly connected to one another in the network (Figure 7A–C). This suggests that, during the specific spatiotemporal window of flower bud morphogenesis, different hormone pathways converge independently onto the central floral regulatory hubs, forming a multi-input, centrally integrated regulatory architecture. Among these, IAA signaling genes (*LAX3/4/5*, *IAA9/17/26/27*, *ARF5*, *GH3.6*, etc.) were highly enriched in co-expression networks with floral induction, integrator (*FT*, *SOC1*), and flower development genes (Figure 7). Moreover, among the hormone-related genes identified as potentially important for flower bud morphogenesis in our study, those involved in IAA biosynthesis/metabolism and signal transduction were the most numerous (31 in metabolism, 45 in signaling; Figure 5), indicating that auxin was broadly active during flower bud morphogenesis in blueberry, not only closely associated with floral induction but also likely regulating floral organ initiation and morphogenesis [44,45,46]. Although direct hormone measurements were not performed, the differential expression patterns of hormone-related genes point to the active involvement of multiple hormone pathways. Future targeted hormone quantification would be valuable to validate these inferred roles.

Regarding transcription factors, a total of 770 differentially expressed TFs from 44 families were identified in this study (Figure 4B). These TFs participate extensively in the regulation of flowering-related genes, constituting a vast transcriptional regulatory network for blueberry flower bud morphogenesis. Among them, MADS-box genes (*FULs*, *AP3*, *PI*, and *SEP2*, etc.) exhibited the highest connectivity in the flower development network (Figure 7C), with *SEP2* and *PI* also showing high connectivity in the interaction network among floral induction, floral integrator, and flower development genes, reinforcing the central role of the MADS-box family as the core regulators. SBP family members *SPL1*, *SPL3*, and *SPL5* were continuously up-regulated and showed high connectivity in the networks, suggesting that they may act as key nodes integrating age signals with downstream floral developmental programs, coordinating flower bud morphogenesis through interactions with hormone signals [14].

### 4.4. A Molecular Regulatory Framework for Bud Tissues During Flower Bud Morphogenesis in Blueberry

**Figure 8:**
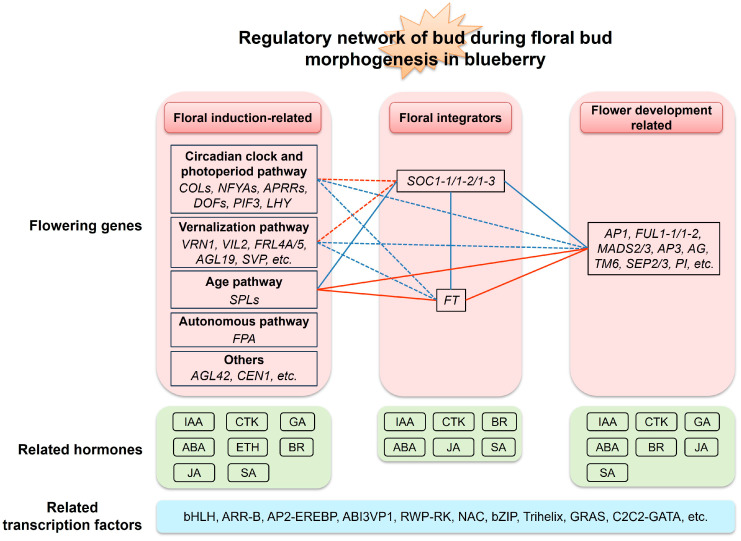
Schematic diagram of the proposed regulatory framework in bud tissues during flower bud morphogenesis in blueberry. Red solid lines indicate positive correlations, blue solid lines indicate negative correlations, red dashed lines indicate predominantly positive correlations, and blue dashed lines indicate predominantly negative correlations.

Integrating the above analyses and co-expression networks, we propose a molecular regulatory framework for bud tissues during the flower bud morphogenesis stage in blueberry (Figure 8). Floral induction genes—predominantly those involved in the circadian clock/photoperiod pathway (*COLs*, *NFYAs*, etc.), vernalization pathway (*VRN1*, *SVP*, etc.), and age pathway (*SPLs*)—integrate diverse endogenous signals and act on the two floral integrators *FT* and *SOC1*, which exhibit a stage-specific functional divergence. During the early-to-middle phase of flower bud morphogenesis, *SOC1* may coordinate with upstream circadian clock/photoperiod and vernalization genes to promote flower bud differentiation. At the late stage, *FT* may synergize with flower development genes to promote floral organ differentiation, while the down-regulation of *SOC1* may prepare for its subsequent functional transition to dormancy regulation. Throughout this process, multiple hormone pathways including IAA, BR, JA, and SA are directly associated with floral induction genes, floral integrators, and flower development genes through their respective biosynthesis/metabolism and signal transduction genes, forming a regulatory network with multiple inputs and concentrated nodes. Other transcription factor families such as bHLH, ARR-B, AP2-EREBP are widely embedded in this network, executing precise transcriptional control. The framework also shows that circadian clock/photoperiod and vernalization pathways are predominantly negatively correlated with flower development genes, whereas the age pathway is uniformly positively correlated with them, reflecting functional differentiation among flowering pathways at the flower development stage.

This framework reveals the molecular architecture of coordinated actions among floral signals, hormonal regulation, and transcriptional cascades in bud tissues during blueberry flower bud morphogenesis, deepening our systematic understanding of the molecular mechanisms underlying blueberry flowering, and providing a valuable theoretical foundation and genetic resources for molecular-assisted breeding. Nevertheless, as this framework is inferred from transcriptomic data, the predicted regulatory relationships presented here await further experimental validation.

## Figures and Tables

**Table 1:** Summary of sequencing data quality and alignment statistics.

Sample	Raw Data (bp)	Clean Data (bp)	Raw Reads	Clean Reads	Clean Reads Ratio	Mapped Reads	Mapping Ratio
B-0W-1	7,419,976,800	7,327,682,467	49,466,512	43,978,502	88.91%	37,367,196	84.97%
B-0W-2	6,256,476,600	6,196,001,254	41,709,844	37,686,344	90.35%	32,247,344	85.57%
B-0W-3	6,448,782,300	6,361,724,281	42,991,882	38,575,662	89.73%	32,720,656	84.82%
B-3W-1	11,226,829,500	11,115,012,059	74,845,530	68,953,288	92.13%	57,408,650	83.26%
B-3W-2	7,359,337,800	7,270,486,799	49,062,252	42,977,374	87.60%	35,008,509	81.46%
B-3W-3	6,938,918,700	6,862,955,456	46,259,458	42,374,588	91.60%	35,643,324	84.11%
B-6W-1	6,067,500,600	6,008,347,892	40,450,004	37,294,194	92.20%	30,810,285	82.61%
B-6W-2	5,852,361,300	5,776,343,879	39,015,742	36,615,918	93.85%	30,776,380	84.05%
B-6W-3	6,112,448,700	6,048,347,056	40,749,658	38,795,096	95.20%	32,776,004	84.48%
B-9W-1	6,486,290,700	6,412,936,086	43,241,938	40,603,158	93.90%	33,413,314	82.29%
B-9W-2	10,427,692,800	10,308,871,540	69,517,952	66,297,884	95.37%	55,342,888	83.48%
B-9W-3	6,612,466,800	6,538,766,245	44,083,112	41,571,486	94.30%	33,977,215	81.73%
B-12W-1	6,056,681,100	5,987,987,790	40,377,874	38,474,544	95.29%	32,563,821	84.64%
B-12W-2	6,080,438,700	6,005,712,366	40,536,258	38,878,432	95.91%	32,454,814	83.48%
B-12W-3	7,548,258,900	7,466,070,993	50,321,726	47,648,084	94.69%	39,812,502	83.56%
B-15W-1	8,354,534,400	8,260,541,941	55,696,896	54,243,192	97.39%	47,679,243	87.90%
B-15W-2	7,282,872,600	7,213,007,865	48,552,484	47,255,772	97.33%	41,587,343	88.00%
B-15W-3	7,401,334,800	7,304,648,081	49,342,232	48,078,514	97.44%	42,160,195	87.69%

Samples are designated as 0W (day of collection, week 0), 3W (week 3), 6W (week 6), 9W (week 9), 12W (week 12), and 15W (week 15), corresponding to the six time points from shoot cessation to bud swelling. -1/-2/-3 denote biological replicates.

## Data Availability

The raw transcriptomic data have been deposited in the China National Genomics Data Center (https://ngdc.cncb.ac.cn/ (accessed on 27 January 2026)) under BioProject accession PRJCA056943 and GSA accession CRA037787.

## Supplementary Materials

The following supporting information can be downloaded at: https://www.mdpi.com/article/10.3390/genes17070837/s1, Table S1: Primer sequences for qPCR. Table S2: The detail information of the node and edge of the co-expression network.
